# The Impact of Financial Education on Stress Among Orthopaedic Surgeons

**DOI:** 10.7759/cureus.58093

**Published:** 2024-04-12

**Authors:** Devan O Higginbotham, Fong H Nham, Daniel R Cavazos, Chaoyang Chen, Steven K Stoker

**Affiliations:** 1 Department of Orthopaedic Surgery, Wayne State University/Detroit Medical Center, Detroit, USA; 2 Department of Orthopaedic Surgery, Granger Medical Clinic, Sandy, USA

**Keywords:** orthopaedic surgery, residency, well-being, stress, financial education

## Abstract

Background: Financial stress has been an increasing area of concern for residents and attendings. The primary goal of this study was to determine the financial education level and differentiate financial outcome measures of orthopaedic surgery residents and attendings.

Methods: A survey of all residents and attendings of the 201 Accreditation Council for Graduate Medical Education (ACGME)-accredited orthopaedic surgery programs in the United States.

Results: Total participation in the study was 118 residents (postgraduate year (PGY) 1-5), three fellows (PGY 6), and 57 attending orthopaedic surgeons. A significant difference existed between average current financial stress scores between residents versus attending (2.32 vs 1.17), but not Doctor of Medicine (MD) versus Doctor of Osteopathic Medicine (DO) attendings (0.96 vs 1.67) and MD versus DO residents (2.25 vs 2.50). There was a significant difference in average future financial stress scores between residents and attendings (1.85 vs 1.44) and MD vs DO residents (1.61 vs 2.25) but no difference between MD vs DO attending (1.31 vs 1.63). Residents’ confidence in financial knowledge compared to college graduates had a significantly negative Pearson coefficient with current financial stress score, while the attending group was not significant.

Conclusions: Orthopaedic residents and attending physicians' financial stress levels are positively correlated with the amount of student debt they hold. Most residents who currently have no personal finance education offered in their residency would likely attend a personal finance course if offered. Decreasing the amount of debt held by residents, increasing their financial knowledge, and helping them develop good financial habits would likely lead to a decrease in financial stress.

## Introduction

Physician wellness stems from complicated multifactorial issues that dramatically affect physicians, patients, and health systems [1,2]. Financial stress and levels of indebtedness have been linked to stress and low levels of wellness in practicing physicians, resident trainees, and academic surgeons, suggesting that these factors may affect physicians across all levels of experience and types of practice [3-6]. Interestingly, most physicians have no formal personal finance education in college or residency training [5,7]. Personal finance education has been associated with increased financial knowledge, improved financial decision-making, and perceived well-being for workers in non-medical fields [8,9]. Additionally, budgeting and saving money are significantly related to the financial well-being of non-medical personnel [10].

The study aims to determine whether increased financial knowledge leads to lower stress levels in orthopaedic surgeons. According to the Medscape 2021 Physician Compensation Report, orthopedic surgeons have the second-highest compensation of all specialties at an average of $511,000 [11]. In spite of this compensation, it is unclear if this group has any better financial knowledge than their medical peers. Further, it is not known if financial stress is caused by factors such as medical education debt and a lack of savings. Currently, there is little information about how financially educated these surgeons are and how resilient they are in handling their financial stresses.

The goal of this study is to evaluate the level of financial education of orthopaedic residents and attending surgeons through a survey mechanism. The primary outcome of this study is to quantify essential financial characteristics of this group, such as their financial education, debt levels, and overall financial stress level. Our primary hypothesis is that poor financial habits due to a lack of financial education along with increased debt burden lead to increased financial stress in both the short term and long term. The secondary outcome is to assess these factors in surgeons at different stages in their careers. Our secondary hypothesis is that there is no correlation between years in training or practice and level of financial education or knowledge and financial habits. The goal of this study is to evaluate these questions in this group and their interplay and to lay the groundwork for understanding financial stresses in physicians on a larger scale.

## Materials and methods

Data was obtained via a survey of orthopaedic surgery residents and attendings who were affiliated with an Accreditation Council for Graduate Medical Education (ACGME)-accredited orthopaedic surgery program in the United States. We identified 201 registered orthopaedic surgery residency programs (representing approximately 3,640 active orthopaedic surgery residents) using the Fellowship and Residency Interactive Database (FREIDA™) database. Residency coordinators at each program were identified from this database and emailed a cover letter describing this study. An electronic link to the anonymous survey was also sent to these coordinators and they were asked to forward this link to the resident and attending surgeons in their respective programs. A reminder e-mail was sent to the program coordinators after one week to encourage participation.

Responses to the survey were accepted between November 23rd, 2020, and December 6th, 2020. Our survey target population was orthopedic residents and fellows in all years of training (postgraduate year (PGY) 1-6) who were in an ACGME-accredited orthopaedic surgery residency program for the academic year (July 1, 2020-June 30, 2021). We also aimed to capture responses from orthopedic attending surgeons who were associated with the respective orthopedic surgery residency programs.

The 32-question survey was based on previously administered surveys that sought to examine financial stresses in other medical specialties [4,5,12,13]. Participation in this study was completely voluntary, and all responses were anonymous as no identifying information was collected. All submitted data were obtained through an online Qualtrics web portal and stored on a secure server affiliated with the authors’ institution. To further encourage participation, all survey participants had the opportunity to enter into a raffle for one of two $100 gift cards. This study was determined to be exempt by the Institutional Review Board at the author's institution (IRB#20-12-3050).

Statistical analysis was performed with the Statistical Package for the Social Sciences (IBM SPSS Statistics for Windows, IBM Corp., Version 26.0, Armonk, NY). Analysis of variance (ANOVA) with post hoc least significant difference (LSD) was used to determine the statistical difference in financial stress score and future financial stress score between resident, fellow, and attending surgeon groups. The correlation between financial stress score and future financial stress score with other measured variables was analyzed. Univariate with post hoc LSD was used to determine the difference between resident and attending surgeons regarding gender. Statistical significance was defined as a p-value < 0.05.

## Results

Demographics

Total participation in the study was 118 residents (PGY 1-5), three fellows (PGY 6), and 57 attending orthopaedic surgeons. The residents and the fellows were aggregated together into a single resident group for the analysis. Basic demographic data about the groups was obtained (Table 1). There were significant differences between age of residents and attendings (30.31 vs 47.49, p-value <0.001). There was a significant age difference between Doctor of Medicine (MD) and Doctor of Osteopathic Medicine (DO) attendings (48.79 vs 40.56, p-value of 0.004). There was no statistically significant difference (SSD) between the age of MD vs DO residents (29.91 vs 31, p-value of 0.063). There were no observed SSDs in gender between residents and attendings (p-value 0.368), MD vs DO residents (p-value of 0.775), and MD vs DO attendings (p-value of 0.688). Regarding the average number of children they have, there was an SSD observed between residents and attendings (0.74 vs 0.64, p-value of 0.648), but SSD between MD vs DO residents (0.74 vs 0.64, p-value of 0.648) and MD vs DO attendings (2.4 vs 2.22, p-value of 0.576). There was an SSD in marital status between residents and attendings (p-value <0.001), but no difference in MD versus DO residents (p-value of 0.312) and no SSD between MD versus DO attendings (p-value of 0.738). There was an SSD in divorce status between residents and attendings (p = 0.045), but no SSD in MD versus DO residents (p-value of 0.584) and no difference between MD versus DO attendings (p-value of 0.109).

**Table 1:** Demographic Data Statistical Significance, p < 0.05 ^a^ t-test; ^b^ Pearson's chi-square test; MD: Doctor of Medicine; DO: Doctor of Osteopathic Medicine

	Residents vs attendings			Residents by degree			Attendings by degree		
	Resident	Attending	p-value	MD	DO	p-value	MD	DO	p-value
	n (%) = 121 (70)	n (%)= 57 (30)		n (%)=76 (63.3)	n (%)=44 (36.7)		n (%)=48 (84.2)	n (%)=9 (15.8)	
Age (Mean ± SD)	Mean ± SD	Mean ± SD	<0.001^a^	29.91 ± 2.65	31.00 ± 3.23	0.063^a^	48.79 ± 10.72	40.56 ± 5.47	0.004^a^
	30.31 ± 2.92	47.29 ± 10.54							
Gender			0.368^b^			0.775^b^			0.688^b^
Male, n (%)	105 (87.5)	47 (82.5)		66 (86.8)	39 (88.6)		40 (83.3)	7 (77.8)	
Female, n (%)	15 (12.5)	10 (17.5)		10 (13.2)	5 (11.4)		8 (16.7)	2 (22.2)	
Professional Degree			0.005^b^	N/A	N/A		N/A	N/A	
MD, n (%)	77 (63.6)	48 (84.2)							
DO, n (%)	44 (36.4)	9 (15.8)							
Number of children	0.7	2.44	<0.001^a^	0.74	0.64	0.648^a^	2.48	2.22	0.576^a^
Marital Status			<0.001^b^			0.312^b^			0.738^b^
Single, n (%)	28 (23.1)	1 (1.8)		20 (26.0)	8 (18.2)		1 (2.1)	0 (0.0%)	
Married, n (%)	76 (62.8)	53 (94.6)		44 (57.1)	32 (72.7)		44 (93.6)	9 (100.0%)	
Other, n (%)	2 (1.7)	0 (0.0)		2 (2.6%)	0 (0.0)		N/A	N/A	
Living with a partner, n (%)	15 (12.4)	2 (3.6)		11 (14.3)	4(9.1)		2 (4.3)	0 (0.0%)	
Divorced			0.045^b^			0.584^b^			0.109^b^
No, n (%)	116 (96.7)	49 (89.1)		75 (97.4)	41 (95.3)		44 (91.7)	5 (71.4)	
Yes, n (%)	4 (3.3)	6 (10.9)		2 (2.6)	2 (4.7)		4 (8.3)	2 (28.6)	

Financial stress

The data from the survey on financial stress is outlined in Table 2. There was an SSD between average current financial stress scores between residents versus attending (2.32 vs 1.17, p-value <0.001), but not between MD versus DO attendings (0.96 vs 1.67, p-value of 0.077) and between MD versus DO residents (2.25 vs 2.50, p-value of 0.299). There was an SSD in average future financial stress scores between residents and attendings (1.85 vs 1.44, p-value of 0.039) and MD vs DO residents (1.61 vs 2.25, p-value of 0.018), but no SSD between MD vs DO attending (1.31 vs 1.63, p-value 0.274).

**Table 2:** Financial Stress of Orthopaedic Surgery Residents and Attendings Statistical significance, p < 0.05 ^a^ t-test; MD: Doctor of Medicine; DO: Doctor of Osteopathic Medicine

	Resident	Attending	p-value	Resident	Resident	p-value	Attending	Attending	p-value
	MD	MD		MD	DO		MD	DO	
	n (%)=121 (70)	n (%)= 57 (30)		n (%)=77 (63.6)	n (%)=44 (36.4)		n (%)=48 (82.7)	n (%)=10 (17.3)	
Current Financial Stress Score (Mean ± Standard Deviation)	2.32±1.29	1.17±1.23	<0.001^a^	2.25±1.36	2.5±1.12	0.299^a^	0.96±1.29	1.67±1.33	0.077^a^
Future Financial Stress Score (Mean ± Standard Deviation)	1.85±1.29	1.44±1.38	0.039^a^	1.61±1.26	2.25±1.28	0.018^a^	1.31±1.36	1.63±1.41	0.274^a^

Resident vs attending financial metrics

There was no difference in the estimated retirement amount between residents and attendings ($4.89 vs $5.46 million, p-value of 0.291) (see Table 3). There was no difference in the planned retirement age between residents and attendings (64.72 vs 63.7, p-value of 0.309). There was a significant difference in the student debt between residents and attendings ($270,833 vs $57,894, p-value <0.001). There was a significant difference in the amount of non-student debt between residents and attendings ($171,717 vs $404,121, p-value <0.001). A higher percentage of attendings (98.2%) had investments versus residents (81.0%) (p-value of 0.002). There was a significant difference in the total investment value for residents versus attendings ($157,500 vs $916,500, p-value <0.001). A higher percentage of residents (68.6%) budget than attending (50.9%) (p-value of 0.022). There was a significant difference between residents and attendings for those who plan on budgeting in the future (p-value < 0.001). A higher percentage of attendings (68.4%) have a financial advisor than residents (32.2%) (p-value < 0.001).

**Table 3:** Financial Metrics of Orthopaedic Surgery Residents and Attendings Statistical Significance, p < 0.05 ^a^ t-test; ^b ^Pearson's chi-square test

	Resident n (%)=121 (70)	Attending n (%)=57 (30)	p-value
Estimated retirement amount ($million dollars) [mean ± standard deviation]	4.89±3.77	5.46±2.98	0.291^a^
Years in practice [mean ± standard deviation]	N/A	13.67±10.69	-
Predicted first-year salary ($)	454,297	N/A	-
Planned retirement age (years)	64.72	63.7	0.309^a^
Student debt ($)	270,833	57,894	<0.001^a^
Non-student loan debt ($)	171,717	404,121	<0.001^a^
Currently have investments?			0.002^b^
No	23(19.0%)	1(1.8%)	
Yes	98(81.0%)	56(98.2%)	
Total value of investments ($) [mean ± standard deviation]	157,500 ± 211,000	916,500 ± 383,500	<0.001^a^
Are currently using a budget?			<0.022^b^
No	38(31.4%)	28(49.1%)	
Yes	83(68.6%)	29(50.9%)	
Plan to budget in future?			<0.001^b^
No	12(9.9%)	20(35.1%)	
Yes	26(21.5%)	8(14.0%)	
Unsure	83(68.6%)	29(50.9%)	
Have a financial advisor?			<0.001^b^
No	82(67.8%)	18(31.6%)	
Yes	39(32.2%)	39(68.4%)	

Correlation between current financial stress score and other factors

There were 121 residents and 57 attendings in the analysis of the current financial stress score and associated factors as outlined in Table 4. Residents and attendings had a significantly positive Pearson correlation coefficient between current financial score and stress about future finance score (Pearson coefficient of 0.384 and 0.761, p-value < 0.001). Residents and attendings had a significantly negative Pearson correlation coefficient between current financial stress score and percentage of gross income saved during residency (Pearson coefficient of -0.342 and -0.22, p-value < 0.001). Residents’ confidence in financial knowledge compared to college graduates had a significantly negative Pearson coefficient with current financial stress score (Pearson coefficient of -0.340, p-value <0.001), while the attending group was not significant (p-value of 0.065). The percentage of gross income saved was negatively correlated with the current financial stress score in the resident group (Pearson coefficient of -0.340, p-value <0.001), while it was not significant in the attending group (p-value of 0.175). Future and current budgets were significantly correlated with higher financial stress scores for residents (Pearson coefficient of 0.195 and 0.187, p-value of 0.032 and 0.04), but budget was not significant for attendings and financial stress scores. The current value of investments was negatively correlated with current financial stress scores for attendings (Pearson coefficient of -0.320, p-value of 0.018), but was not significantly correlated to stress for residents. The current amount of student debt was positively correlated with current financial stress scores for attendings (Pearson coefficient of 0.316, p-value of 0.017), but was not significantly correlated to stress for residents. The age of planned retirement was positively correlated with current financial stress scores for attendings (Pearson coefficient of 0.272, p-value of 0.041), but was not significantly correlated to stress for residents. The educational degree was positively correlated with current financial stress scores for attendings (Pearson coefficient of 0.278, p-value of 0.036), but was not significantly correlated to stress for residents. Current investments, current age, budget methods, divorce status, personal finance education in residency, money needed to retire, current number of kids, willing for personal finance education in residency, budget tracking methods, expected earnings first year as an attending, formal personal finance education, or non-student loan debt were not significantly correlated with current financial stress scores for both residents and attendings.

**Table 4:** Correlation Between Current Financial Stress Score and Other Factors Statistical Significance, p < 0.05

		Resident (n=121)		Attending (n=57)		Statistical explanation
Current Financial Stress Score versus	Statistical method	Correlation Coefficient	p-value	Correlation Coefficient	p-value	Bivariate correlate analysis (two-side-tailed)
Stress About Future Finance Score	Pearson	0.384	<0.001	0.761	<0.001	Future stress score increased with current stress score among both residents and attendings
Percentage of GROSS income saved during residency	Pearson	-0.342	<0.001	-0.22	<0.001	More saving, less stress score
Confidence of financial knowledge compared to other college graduates	Pearson	-0.340	<0.001	-0.246	0.065	Higher confidence yielded less stress score among both residents and attendings
Percentage of GROSS income saved currently?	Pearson	-0.253	0.005	-0.182	0.175	Less money saved yielded a higher stress score among residents
Future Budget	Spearman	0.195	0.032	0.048	0.722	Having future budget yielded higher stress score among residents
Current Budget	Spearman	0.187	0.04	0.009	0.947	Having current budget yielded higher stress score among residents
Current value of all of your investments	Pearson	-0.15	0.104	-0.320	0.018	Higher investment yielded less stress score among attendings
Current student loan debt	Pearson	0.156	0.108	0.316	0.017	Higher current student load yielded higher stress score among attendings
Current investments	Spearman	-0.128	0.16	0.149	0.268	Current stress score was not significantly associated with current investment among both residents or attendings
Current Age	Pearson	0.113	0.216	-0.171	0.204	Current stress score was not significantly associated with current age among both residents or attendings
Budget methods	Pearson	0.112	0.222	0.137	0.311	Current stress score was not significantly associated with budget methods among both residents or attendings
Age planning on retiring	Pearson	0.091	0.323	0.272	0.041	Current stress score was not significantly associated with planned retiring age among both residents or attendings
Education degree	Spearman	0.079	0.391	0.278	0.036	Current stress score was not significantly associated with educational degree for residents
Divorced	Spearman	0.075	0.414	-0.1	0.466	Current stress score was not significantly associated with divorce for both residents or attendings
Personal finance education in residency	Spearman	0.07	0.443	-0.172	0.2	Current stress score was not significantly associated with personal finance education in residency for both residents or attendings
Money needed to retire	Pearson	0.068	0.469	-0.088	0.523	Current stress score was not significantly associated with money needed to retire among both residents or attendings
Number of Kids	Pearson	0.061	0.51	-0.182	0.183	Current stress score was not significantly associated with number of kids for both residents and attendings
Willing for personal finance education in residency	Spearman	-0.041	0.72	-0.009	0.95	Current stress score was not significantly associated with willing for personal finance education in residency among both residents or attendings
Budget tracking methods	Spearman	0.03	0.745	-0.154	0.252	Current stress score was not significantly associated with budget tracking methods for both residents or attendings
Expected earnings in first year as an attending	Pearson	0.015	0.87			Current stress score was not significantly associated with the expected first-year earnings as attendings
Formal personal finance education while in college or during residency	Spearman	0.014	0.883	-0.201	0.133	Current stress score was not significantly associated with formal personal finance education while in college or during residency among both residents or attendings
Non-student loan debt	Pearson	-0.011	0.933	0.233	0.08	Current stress score was not significantly associated with non-student loan debt among for both residents or attendings

MD vs DO comparison

The data for the comparison between MD and DO participants is outlined in Table 5. Participation varied with 38.4% (48) of attending MD responses, 17.0% (nine) of attending DO responses, 61.6% (77) resident MD responses, and 83.0% (44) resident DO responses (p-value < 0.005). Financial stress scores were higher for DO responses about current (2.34 vs 1.74) and future (2.17 vs 1.55) finances (p-value 0.003). There was no difference in previous former finance education in MD versus DO participants (p-value of 0.256). Residencies that offered personal finance education were 26.4% for MD responses and 22.6% for DO responses (p-value of 0.589). If personal finance education during residency was offered, 88.8% of MD responses would attend compared to 70.0% of DO responses (p-value < 0.001). There was no significant difference in the average financial knowledge between MD and DO participants (0.42 vs 0.34, p-value of 0.651). There was no difference between MD and DO participants in the gross income saved during residency (13.16% vs 13.30%, p-value of 0.941). There was no difference between MD and DO participants in gross income saved now (22.32% vs 18.02%, p-value of 0.081) and income saved in the future (30.8% vs 28.2%, p-value of 0.122). There was no difference in the percentage of MD and DO participants currently budgeting (p-value of 0.575). There was no difference in the percentage of MD and DO participants currently investing (p-value of 0.944). There was a significant difference in the value of investments between MD and DO participants ($509,000 vs $248,000, p-value < 0.001). There was a significant difference in the amount of student loans between MD and DO participants ($129,500 vs $303,000, p-value < 0.001). There was a significant difference in the amount of non-student loan debt between MD and DO participants ($308,810 vs $210,270, p-value of 0.049). There was no difference in the usage of a financial advisor (p-value of 0.798).

**Table 5:** MD vs DO Comparison Statistical Significance, p < 0.05 ^a^ t-test; ^b^ Pearson's chi-square test; MD: Doctor of Medicine; DO: Doctor of Osteopathic Medicine

	MD	DO	P-values
Position			
Residents, n (%)	77 (61.6)	44 (83.0)	0.005^b^
Attendings, n (%)	48 (38.4)	9 (17.0)	
Stressed about CURRENT finances (0-4)	1.74±1.34	2.34 ± 1.11	0.003^a^
Stressed about FUTURE finances (0-4)	1.55±1.24	2.17 ± 1.23	0.003^a^
Formal personal finance education in college or residency, n (%)			0.256^b^
No	93 (74.4)	35 (66.0)	
Yes	32 (25.6)	18 (34.0)	
Residency offer personal finance Education? n (%)			0.589^b^
No	92 (73.6)	41 (77.4)	
Yes	33 (26.4)	12 (22.6)	
If offered in residency, would you participate in personal finance? n (%)			<0.001^b^
Yes	79 (88.8)	28 (70.0)	
No	4 (4.5)	12 (30.0)	
Maybe	6 (6.7)	0 (0.0)	
Financial knowledge compared to college graduates (0-5)	0.42 ± 1.12	0.34 ± 1.14	0.651^a^
Gross Income saved during residency (%)	13.16 ± 11.40	13.30 ± 11.81	0.941^a^
Gross Income saved NOW (%)	22.32 ± 15.28	18.02 ± 14.72	0.081^a^
Gross Income saved FUTURE (%)	30.8 ± 11.65	28.2 ± 9.5	0.122^a^
Currently budgeting, n (%)			0.575^a^
No	48 (38.4)	18 (34.0)	
Yes	77 (61.6)	35 (66.0)	
Have investments? n (%)			0.944^b^
No	17 (13.6)	7 (13.2)	
Yes	108 (86.4)	46 (86.8)	
Value of Investment ($)	509,000 ± 485,000	248,000 ± 341,500	<0.001^a^
Student loan debt ($)	129,500 ± 138,000	303,000 ± 169,000	<0.001^a^
Non-student loan debt ($)	308810 ± 343807	210270 ± 197210	0.049^a^
Financial advisor, n (%)			0.798^b^
No	71 (56.8)	29 (54.7)	
Yes	54 (43.2)	24 (45.3)	

## Discussion

Financial stress is multifactorial with significant economic impact in the current healthcare system. Financial stress has been previously linked to physician well-being and prosperity in previous publications [3-6]. While other surveys of orthopaedic surgery trainees have shown low levels of financial literacy and financial education, this was the first study to quantify the levels of financial stress in both trainees and attendings in orthopaedic surgery [14,15]. This study demonstrates that the level of financial education and debt may contribute to stress in orthopedic surgery residents and attendings. Our findings highlight a negative correlation between future financial stress and financial knowledge. For attending physicians, the amount of debt a physician carries is proportional to the amount of current and future financial stress they feel. For resident physicians, there is a direct correlation between the debt amount and their current/future financial stress levels, but the correlation was only significant for future debt. This may be attributable to income-based repayment plans many residents enroll in allowing for a proportion of their income towards student loans while in residency, and thus presenting with lower current financial stress and higher future stress. Our results also show that as attending physicians’ total investments increase, their financial stress scores decrease. Decreasing the amount of debt a physician carries and increasing the amount of investments held leads to decreased financial stress and should lead to decreased physician disengagement [4,5]. Improvement in financial literacy through education in the training and work environment can positively influence financial planning behaviors and decisions [16].

Perceived financial knowledge showed a negative correlation with how a participant felt compared to their non-medical peers and with their level of financial stress. As a participant increased in perceived knowledge, financial distress decreased. Previous studies in non-medical fields have shown individuals who participated in formal personal finance education have higher objective levels of financial knowledge as well as perceived financial knowledge [8]. This suggests that residency-based personal finance education would increase perceived financial knowledge and, in doing so, subsequently decrease future financial stress. This study also showed that residency-based personal finance classes would be extremely popular, with 77% of residents saying they would attend the classes and another 20% saying they might attend the classes. This strengthens the argument from previous publications that personal finance education should be a required part of the residency curriculum [12,14]. As resident physicians obtain greater levels of financial knowledge, it is expected their financial stress levels will decrease and hopefully cause a better outlook regarding financial success.

A survey of college students reported both good and bad financial habits were linked to financial well-being [9]. Our study demonstrates a similar correlation between good financial behavior and financial stress such as the role of budgeting in decreasing the future financial stress level. Resident and attending physicians who have a budget and live by it had the second lowest financial stress score. The physicians who currently do not have a budget, but plan on doing it in the future had the next highest financial stress score. Those who reported having a budget but not following it had the highest stress score of all groups. These findings suggest that cultivating effective financial habits, such as creating and sticking to a budget, can potentially reduce future financial stress. Interestingly, the study also found that individuals who do not budget or plan to budget experience reduced stress levels. This effect is not well understood but may be related to a lack of understanding of their financial situation. Surprisingly, having children during residency or as an attending did not contribute to an increase in financial stress.

This study shows a statistically significant correlation between the type of medical degree obtained (MD vs DO) and the amount of student debt that a resident holds. DO residents have nearly two times the student debt of their MD counterparts, half the value of total investments, while not showing significantly more non-student debt. The current and future financial stress scores of DO residents are significantly higher than those of their MD counterparts as well. Although a direct correlation cannot be established from the data, there is a clear connection between the amount of student debt and the higher levels of future financial stress experienced by DOs compared to MDs.

This study has several limitations. First, because we utilized a voluntary survey mechanism to obtain data, it is possible that participation might have been affected by the level of one’s interest in financial matters. It is possible that certain metrics, such as financial education, debt levels, and ability to budget, may have been poorly estimated due to the fact that the survey respondents may have felt more financially skilled. This could potentially lead to an overestimation of the overall financial well-being of the orthopaedic surgery population. Second, since this was a survey of only orthopedic surgery residents and attendings, these findings may not be generalizable to physicians in other specialties. Third, the attending physicians who were surveyed have ties to academic institutions and thus may not be representative of orthopedic attending physicians, i.e. those in private practice. Last, as a financial incentive was offered, this may have disproportionately encouraged some physicians to participate.

## Conclusions

Financial stress has been previously linked to physician well-being. Orthopaedic surgery residents and attending physicians' financial stress levels are positively correlated with the amount of student debt they hold. It is likely that cultivating effective financial habits and increasing financial knowledge among physicians would lead to decreased disengagement among them. Most residents would likely attend a personal finance course if it were offered. Reducing the amount of debt held by residents, enhancing their financial knowledge, and helping them develop effective financial habits can lead to a reduction in financial stress and an increase in overall well-being.
